# Food compensation: do exercise ads change food intake?

**DOI:** 10.1186/1479-5868-8-6

**Published:** 2011-01-28

**Authors:** Ellen van Kleef, Mitsuru Shimizu, Brian Wansink

**Affiliations:** 1 Wageningen University, Marketing and Consumer Behaviour Group, Hollandseweg 1, 6706 KN Wageningen, the Netherlands; 2 Cornell University, Food and Brand Lab, 110 Warren Hall, Ithaca NY 14853, USA

## Abstract

**Background:**

Past research has shown that promotional messages such as food advertising influence food consumption. However, what has gone largely unexplored is the effect of exercise advertising on food intake. This study experimentally tested the effects of exposure to exercise commercials on food intake at a lunch meal as compared to the effects of control commercials.

**Methods:**

Prior to eating lunch, 125 participants (71 women, 54 men) watched 8 commercials, either all related to exercise or fitness (n = 67) or neutral products (i.e. car insurance) (n = 58). The meal consisted of a pasta dish with tomato sauce, salad and chocolate pudding. The post-lunch questionnaire included questions about body mass index, exercise habits, motivation and dietary restraint.

**Results:**

Participants exposed to exercise commercials reduced their caloric intake by 21.7% relative to the control condition. Additionally, watching exercise messages increased the perceived healthiness and liking of the meal. Although exercise habits and intentions did not moderate the effect of commercial condition on food intake, we also found that this intake reduction was driven by participants with higher body mass index levels.

**Conclusions:**

These results imply that exercise messages may serve as a reminder of the link between food and physical activity and affect food consumption. It also highlights the need for increased awareness that these messages have powerful influences not only on exercise behavior, but also on closely related behaviors such as eating.

## Background

Many health benefits are associated with physical activity such as a positive mental health [1] and a lower risk of chronic diseases, for example coronary heart disease, diabetes, certain types of cancer and osteoporosis [2]. However, due to the obesity crisis and rapidly declining physical activity levels in many countries world wide, promoting physical activity has been identified as a major public health priority [3]. Both a reduction of food intake and an increase in physical exercise are seen as highly necessary and complementary routes in the battle against overweight and obesity [4]. Numerous initiatives, (inter)national guidelines and campaigns have emphasized the significant role physical activity plays in maintaining good health and preventing diseases. Unfortunately, these efforts have so far not translated into increased physical activity levels. The effectiveness of physical activity promotion has been the topic of extensive research [5,6]. A key result of these studies is that although many people are aware of the health benefits of exercise, promotional media campaigns have little or no impact on exercise behaviour [7].

Past research has shown that consumption decisions are influenced by promotional messages, such as food advertising [8,9]. What has been largely unexplored is the effect that exercise commercials have on immediate food consumption and food evaluations. A recently conducted study showed that food consumption can be influenced by exercise promotion messages [10]. Albarracin, Wang and Leeper [10] conducted two laboratory experiments to study changes in food intake following exercise messages. In the first experiment, participants were either exposed to five exercise promotion posters or similar looking control posters with the supposed objective of rating the appeal and likely efficacy of the ads. Immediately after this task, participants evaluated twenty raisins in a taste test, with the instruction to eat as many as they wanted. Results show that recipients of the exercise message ate an average 18 calories relative to about 12 calories in the control condition. A similar significant increase in food consumption of about 20% was observed in a second experiment where participants were subliminally exposed to either action words (i.e. 'active' or 'go') versus control words (i.e. 'pear' or 'moon').

To examine the generalizability and stability of this effect, more research is needed, because exposure to this type of messages with a resulting increase in food intake could potentially backfire against people, particularly those who exercise to manage their weight. Although real-life commercials may vary in persuasiveness and mood arousal, we used exercise related commercials instead of exercise promotion posters used by Albarracin and colleagues [10] in our attempt to increase the external validity of the study. Therefore, the main research aim of the present study is to test the effects of exposure to exercise commercials on actual food intake. Besides testing the effect of exercise commercials on the intake of foods during a meal following watching exercise commercials, an additional aim of the present study is to examine how individuals' characteristics (i.e. weight status, exercise habits and intentions) play a role.

### Theoretical background

People typically process promotional messages, such as exercise commercials, with low levels of involvement and do not always cognitively elaborate on the meaning [11]. According to the research on priming, the effect of advertising on behaviour may even occur outside of people's conscious awareness by subtly reminding them of the associations we should have of the products promoted. Priming or non-conscious activation of social knowledge structures has been extensively studied over the past decades [12]. Research on how people set and pursue their goals has shown that mental representations of goals can also be triggered by environmental cues without a consciously made choice such that subsequent behaviour is then guided by these goals [13,14]. For example, priming hedonic consumption goals tends to intensify the desire for hedonic foods. Harris, Bargh and Brownell [8] present evidence of an automatic causal link between food advertising as a prime and greater snack consumption. They found that food advertising increased food consumption and these effects were not related to hunger or other conscious influences. Similarly, studies have shown that observing someone engaging in an action or imagining action leads to an increased tendency to perform that behaviour oneself [15,16]. In explaining the behavioural effects of priming, the ideomotor theory proposes that behaviour follows automatically from the activation of relevant mental contents. In other words, merely thinking about doing something automatically makes it more likely that you will perform the action [17].

Basically, all kinds of psychological concepts and processes (e.g. stereotypes, traits, social norms, goals) can be primed or put into motion without conscious awareness [12]. Relevant in this study is the recently proposed model of general action and inaction goals by Albarracin and colleagues [18]. Action goals are goals with end states at the extreme of the continuum of activity level. Once set, people may search for and engage in variety of behaviours to reach the end state of high activity. General states of action and inaction can also be activated by primes. For example, action goals can be activated by exposure to action words (e.g. 'act'), but as well by exposure to exercise promotion messages, as in the study examining the effect of exercise promotion posters [10,18]. Albarracin and colleagues [10] state that motivational mechanisms associated with the use of action words and images are likely to be automatically unfold when exercise-promotion messages are presented. For example, words such as 'active' and 'go' may produce a generalized desire for motor output that can be satisfied with eating [10]. Hence, similar to the study of Albarracin and colleagues [10], exposure to exercise commercials may also trigger automatic eating of available foods. Therefore, our primary hypothesis is that people would have a significantly higher calorie intake after watching exercise commercials compared to watching neutral commercials.

Furthermore, we expect that the effect of exercise messages will be moderated by individuals' weight status. That means, in stead of triggering the tendency to eat, people with higher body mass index levels will restrain themselves from eating too much. This expectation is based on findings in the literature on stereotype priming. Advertising typically strongly reflects current stereotypes in a society to bring the message to the public. There is considerable evidence that primed stereotypes elicit corresponding behaviour in the perceiver [16]. Two categories of effects of priming stereotypes can be distinguished. That is, people's behaviour can become consistent with the primed stereotype (assimilation effect) or inconsistent with the primed stereotype (contrast effect) [17]. In their review of the literature, Wheeler and Petty [17] report that the majority of published experiments demonstrated that people assimilate their behaviour to the activated stereotype. Under some conditions, however, they make evoke contrast effects. Contrast effect are more likely to occur when the primed stereotype is very dissimilar, concrete and relevant in relation to self-perceptions [16]. Since the primed stereotype in a typical exercise commercial involves attractive, confident and skinny individuals, a contrast effect is more likely to be expected than an assimilation effect for people with higher body mass index (BMI) levels. Moreover, the primed stereotype in this type of commercial is self-relevant as many overweight individuals are usually dissatisfied with their weight and try to loose this weight. Exercise is associated with trying to loose weight, particularly for women [19]. This is consistent with the model of general action and inaction goals, which proposes that general action goals are tied to specific goals. Supporting this argument, lowering food intake is seen as the better way to achieve the goal of weight loss than physical activity alone or a combination of the two [20]. People realize that although exercise burns off calories, a lot of exercise is needed to burn off a relatively small number of calories [21]. Moreover, individuals trying to lose weight have not adopted regular physical activity as part of their weight loss practice [22]. An Australian study found that overweight young women were less likely than normal weight women to see leisure time physical activity as being feasible. It is likely that embarrassment, lack of confidence and physical discomfort are key factors explaining this [23]. In sum, the effects of activating the exercise node in memory of people with higher body mass index levels predict a contrast effect for caloric intake rather than the assimilation effect predicted for normal weight individuals.

In sum, we predict that exercise commercials increase caloric intake of a following meal. In contrast, we also expect that individuals with higher body mass index levels reduce their caloric intake. We test these predictions in a laboratory experiment in which we randomly assigned participants to two experimental conditions (exercise commercials versus neutral commercials).

## Methods

### Design

Participants were randomly assigned to either an exercise commercial condition or a control commercial condition. Participants watched commercials first and then served themselves food one by one. Two additional conditions were created which were watching a television cartoon after serving oneself food (so during consumption) or not watching a cartoon. The idea of this manipulation was that television watching might impact food intake. Identical procedures were followed during the experiment where participants watched a cartoon and the experiment where participants did not watch a cartoon while eating. The amounts of total food and individual foods consumed did not differ between the two experiments (all *F*s < 2, *p*s > 0.24). Therefore, results from both conditions were combined to increase the statistical power of the analyses.

### Participants

Participants were 128 Cornell University undergraduate students (73 women, 55 men) who participated in exchange for money or extra course credit. The study was approved by the Institutional Review Board.

### Procedure and materials

Participants signed up for the study which was held during lunch break (between 11.30 am and 1.30 pm). Participants were welcomed and seated at a table. The first part of the experiment consisted of an advertising evaluation task in which participants were either exposed to a series of eight exercise commercials (4 minutes and 57 seconds in total) or a control condition of eight commercials (4 minutes and 59 seconds in total) that did not refer to foods or exercise (i.e. car insurance, home appliance, pet adoption program). Instead of public health campaign commercials, we selected commercials of exercise equipment (i.e. running shoes) and services (i.e. fitness centre, fitness program) because these commercial messages dominate the messages that people see regarding physical activity and public health campaign commercials are relatively infrequent [24]. Both men and women were featured in these commercials which were actively engaged in sports. Care was taken that no explicit weight management or weight loss message was included in the commercials.

To shield the purpose of the manipulation, the evaluation of the commercials was presented as an advertising study. To support the cover story, after each pair of commercials, participants rated the commercials on some attributes and indicated which one they liked the most. They also responded to items which all started with 'watching these commercials made me...' followed by: happy, hungry, feel somewhat guilty, feel athletic, feel relaxed, feel active, healthy and in good shape. Each item is scored on a scale from 1 (strongly disagree) to 7 (strongly agree). A 7-point Likert type scale was included which asked participants to indicate the likelihood of going to the gym the coming week (1 = very unlikely and 7 = very likely). Gym was selected as this is a common form of physical activity among college students.

Immediately after the commercial evaluation task, participants were instructed to line up for the food buffet. The meal consisted of a pasta dish with a tomato sauce, a salad, and a chocolate pudding. Chocolate was selected as this is typically seen as an indulgent calorie rich food, which might activate self-control mechanisms. In addition, salad dressing (light or regular), cheese and drinks (Regular Coke, Diet Coke or water) were available. At the buffet table, pasta, salad and pudding were placed on scales. The scales were hidden for participants by large table cloths. All scales had longer cords leading to a digital display that was placed out of sight of participants. Research assistants noted the weight of the scale before a participant spooned food out of the bowl. When the participant finished spooning food out of the bowl, the weight was again noted. The total amount of food served was calculated by subtracting the second measurement from the first. After serving, the participant sat down at a table to eat. When done, the tray with leftovers was taken away and a second questionnaire was handed to the participants. Leftovers were measured out of sight of participants to calculate the amount of food consumed. No second servings were allowed. Salad dressing, type of drink and cheese taken with the meal were not weighted but research assistants identified the choices participants made.

A few times, research assistants were not able to note the one or more weights, for example because a participant handed the serving spoon to next participant without putting it down in the bowl. As a result, nine data points (3 concerning salad; 2 concerning pasta and 4 concerning pudding) were missing. Grams were converted into calories (pasta was 75 calories per 100 grams, salad was 14 calories per 100 grams and chocolate pudding was 149 calories per 100 grams).

#### Post-meal questionnaire

After eating their lunch, participants filled out a questionnaire with items related to their liking of the foods, their moods and several scales. We measured how participants felt on a four-item, seven-point mood scale [25], anchored by 'sad/happy', 'bad mood/good mood', 'irritable/pleased' and 'depressed/cheerful'. We also included some mood descriptors from the PANAS scales [26]: active, strong, proud, guilty as they seem relevant in relation to our study objective.

As high self-esteem is associated with healthy behaviours such as physical activity [27], short-lived (i.e. state) changes in self-esteem were measured according the scale of Heatherton and Polivy [28]. Some items were adapted to better fit the purpose of this study (see Appendix 1 for items). Each item is scored on a scale from 1 (strongly disagree) to 7 (strongly agree). The items were preceded by the phrase 'Answer these questions as they are true for you right now'.

Furthermore, participants self-reported their height and weight, which were used to calculate body mass index (BMI). As a manipulation check, it was asked how much effort participant put in the task, the time since they had their last food and how familiar, appealing and convincing they rated the shown commercials. Participants were asked about their physical activity habits by the Godin Leisure Time Exercise Questionnaire (GLTEQ) [29]. The three item GLTEQ assesses the frequency of performed strenuous exercise, moderated exercise and mild exercise for at least 15 minutes in a typical week. Total weekly leisure activity is calculated by summing up the products of the separate components as follows: Total exercise = (frequency of mild exercise × 3) + (frequency of moderate exercise × 5) + (frequency of strenuous exercise × 9). The GLTEQ has been found to have good 2-week test-retest reliability and construct validity [30].

As people's motives to exercise vary [31,32], exercise motivation was assessed by a modified version of the Exercise Motivation Scale [33]. A seven-point Likert-type response format ranging from 'strongly disagree' to 'strongly agree' was used for all items (see Appendix 1 for overview of items and reliability). Perceived exercise behavioural control was included to measure an individual's self-confidence in being able to engage in physical activity, based on the scale of Armitage [34]. Dietary restraint was assessed as possible explanatory variable and measured with a 10-item scale from Polivy, Herman and Warsh [35]. reliability of this scale in the current study was α = 0.73. The Restraint scores ranged from 4 to 29 with a mean of 14.02 (SD = 5.5).

### Data analysis

Using analyses of variance (ANOVAs) we first checked whether there were differences between commercial condition (exercise versus control) and the time since participant had last food, BMI, exercise habits, commercial evaluations and exercise motivation and exercise behavioural control to examine whether our randomization was successful. To test the effect of commercial condition on mood, food intake and lunch evaluations we used ANOVAs. Additional analyses were conducted to test for the interaction effects of commercial condition and possible moderator variables (i.e. weight status, dietary restraint, exercise habits, exercise intentions). Moderated multiple regression analysis was carried out. Simple slope analysis to compare non-standardized betas was done if a moderating effect was identified.

## Results

### Manipulation checks

Even though the experiment was announced to be not suitable for vegetarians (the pasta dish served contained chicken), two female participants indicated to be vegetarian and were removed from the dataset. The screening procedure led us also to remove one male participant whose exercise pattern deviated strongly from all other participants. His weekly exercise activity, measured by the GLTEQ deviated more than five standard deviations from the mean exercise score in the entire sample, suggesting that this participant could be a professional athlete, leaving 125 (71 women, 54 men) in the analysis. The average of the final sample was 20.5 years (SD = 5.0) and a mean BMI of 22.7 kg/m^2 ^(SD = 3.3).

Time since last food intake did not differ between conditions. There were also no differences in the effort put into the task (all Fs < 1). All commercials were equally familiar and appealing. However, participants found the control commercials more convincing (F(1,123) = 10.54, p < 0.01). There were also no differences in BMI and exercise habits as measured by the GLTEQ between the groups. In addition, there were no differences across conditions in the likelihood of going to the gym the coming week and ratings of exercise motivations and exercise behavioural control (all Fs < 1).

### Message effects on amount of foods consumed

When all three foods are considered together, results showed that participants consumed 299.5 grams of food on average (188.0 grams of pasta, 47.3 grams of salad, and 69.1 grams of pudding; standard deviations 97.8, 24.5, 56.7 and 119.3 for pasta, salad, pudding and total foods, respectively)(Table 1). After watching the exercise commercials, participants served themselves less foods in total (Table 2), resulting in a lower overall food and calories intake (F = 5.54, p = 0.02 and F = 8.67, p < 0.01). In particular, participants being exposed to exercise commercials reduced their caloric intake of the meal by 21.7% relative to the control condition. There were no differences across the two conditions in the type of drink and salad dressing participants took (both ps > 0.22). Watching the control versus the exercise commercials did not lead to other choices of putting cheese on the meal (p = 0.36).

**Table 1:** Mean (SD) of total food consumption (pasta, salad and pudding in grams and calories)

	Control commercials	Exercise commercials	p-value
Food served in total (grams)			
Total	364.67 (121.77)	316.40 (103.79)	0.02
Men	401.04 (131.47)	345.04 (114.22)	0.11
Women	339.00 (109.19)	294.92 (91.02)	0.07

Food eaten in total (grams)			
Total	324.32 (126.72)	276.95 (108.25)	0.03
Men	360.13 (134.35)	314.00 (116.95)	0.20
Women	298.27 (116.05)	249.17 (95.52)	0.06

Food eaten in total (calories)			
Total	276.87 (122.76)	216.87 (100.14)	<0.01
Men	317.65 (128.01)	246.37 (92.71)	0.03
Women	247.21 (111.52)	194.75 (101.01)	0.04

**Table 2:** Mean (SD) of effects of commercials

	Control commercials (n = 58)	Exercise commercials (n = 66)	p-value
*Watching these commercials made me...*			
happy	4.64 (0.97)	4.61 (1.11)	0.87
hungry	2.72 (1.69)	3.21 (1.44)	0.09
feel somewhat guilty	2.88 (1.72)	3.68 (1.92)	0.02
feel athletic	2.41 (1.31)	4.71 (1.60)	<0.01
feel relaxed	4.48 (1.27)	3.39 (1.21)	<0.01
feel active	2.66 (1.53)	4.68 (1.57)	<0.01
healthy	2.66 (1.45)	4.18 (1.55)	<0.01
in good shape	2.59 (1.39)	3.86 (1.63)	<0.01
Physical performance satisfaction	5.11 (1.05)	4.99 (1.25)	0.56
Body and weight satisfaction	4.45 (1.23)	4.55 (1.26)	0.65
*Lunch evaluations*			
'I like the food I ate'	4.97 (1.31)	5.41 (1.11)	0.04
'The pasta was very good'	4.93 (1.32)	5.14 (1.40)	0.41
'The chocolate pudding was very good'	4.79 (1.47)	5.18 (1.60)	0.17
'The salad was very good'	4.04 (1.41)	4.76 (1.35)	<0.01
'The salad dressing was very good'	4.21 (1.52)	5.03 (1.31)	<0.01
'The lunch was really healthy'	3.93 (1.24)	4.45 (1.25)	0.02
'At this moment I feel full'	5.05 (1.48)	5.28 (1.43)	0.39
'I could not eat another bite'	3.47 (1.63)	3.50 (1.80)	0.93
*Mood*			
Sad - happy	5.12 (0.94)	5.42 (0.88)	0.07
Bad mood - good mood	5.03 (1.18)	5.32 (1.18)	0.19
Irritable - pleased	4.88 (1.17)	4.97 (1.53)	0.72
Depressed - cheerful	4.93 (1.06)	4.98 (1.30)	0.80
Inactive - active	4.21 (1.39)	4.44 (1.60)	0.39
Weak-strong	4.55 (1.19)	4.76 (1.42)	0.39

The correlation between the amount of food participants served themselves and the amount they consumed was high (r(120) = 0.94, p < 0.001). The average difference between what people served themselves and what they ate was low (M = 40.6 grams), which implies that participants ate almost all the food they served themselves. As a result, all analyses of the amount consumed showed identical results as with the analyses of the amount served. Therefore, from now we only report the analyses of the amount of foods consumed.

### Commercial effect on reported feelings, self-esteem, lunch evaluations and mood

Watching the exercise commercials made participants feel less relaxed, more athletic, healthy and in good shape than watching the control commercials (all *p*s < 0.05). Participants' mood was not affected, although there was a marginal effect on the dimension 'sad-happy' in that the exercise commercials made participants feel slightly happier (p = 0.07). The exercise commercials also resulted in a marginally higher rating of feelings of hunger (p = 0.09). Regarding the lunch evaluations, there was a significant difference in participant ratings of the healthiness of the meal between the two conditions (p = 0.02). Participants in the exercise commercial condition rated the lunch as healthier compared to participants in the control condition. The food was also more liked in the exercise message condition, particularly the salad and salad dressing (all ps < 0.05). Despite differences in overall energy intake, ratings on hunger and fullness (i.e. 'at this moment I feel full' and 'I could not eat another bite') did not differ by condition (p = 0.39 and p = 0.93 respectively). It was checked whether the vitality feelings after watching the exercise commercials and healthiness ratings of the lunch mediated the relation between commercial exposure and food intake, but this was not the case. It was also checked whether food intake mediated the relation between commercial exposure and healthiness ratings of the lunch, as it may be that the on average smaller portions consumed are perceived as healthier. However, this was also not the case. This suggests that effects of commercial exposure on feelings after watching the commercials and food intake involve different processes.

### Weight status

A possible interaction between message condition and weight status was examined by using a moderated regression analysis, as BMI is a continuous variable [36,37]. First, BMI scores were mean-centered to make the zero a meaningful value so that the intercept in the regression becomes interpretable. The key dependent variable was the total caloric intake. Condition was coded as a dummy variable equivalent to one if the participant was exposed to the exercise commercials and zero if the participant was exposed to the control condition. At first, a regression equation with only condition and BMI as predictors was run. In this model, the beta coefficient for BMI is not statistically significant (unstandardized beta = 2.03, p = 0.51), but condition is statistically significant (unstandardized beta = -59.40, p < 0.01). In a second regression model, condition, mean-centered BMI and the interaction between condition and BMI were included as independent predictors. The beta coefficient of the interaction term (unstandardized beta = -14.95, p = 0.02) is statistically significant from zero. The addition of this product term resulted in an R^2 ^change of 0.045 (F(1,116) = 5.96, p = 0.02). This result shows the presence of a moderating effect explaining 4.5% of variance in caloric intake above and beyond the variance explained by only BMI and condition.

**Figure 1:**
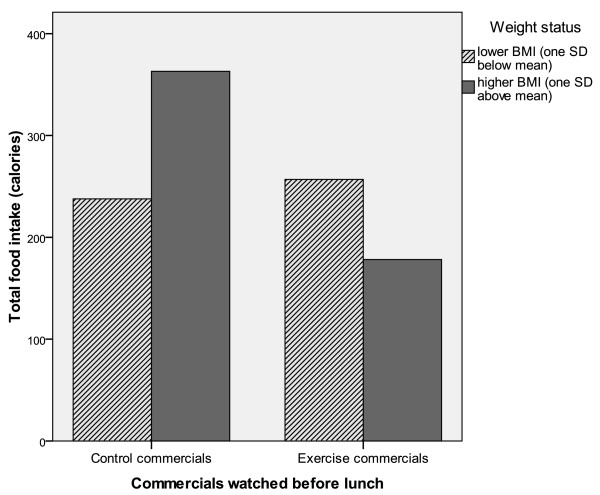
Interaction of weight status by message condition on the total amount of calories consumed

The significant interaction was investigated further with simple slope analysis to assess whether these slopes were different from zero. Results show that for participants with a higher BMI (1 SD above the mean) the slope was significant different from zero (β = -108.57, t(116) = -3.82, p < 0.001), but the simple slope for participants with lower BMI (1 SD below the mean) was not significant different from zero (β = -8.67, t(116) = -0.30, p = 0.77). To illustrate the effect of the interaction coefficient, Figure 1 below shows the differences between participants with low and high BMI at plus and minus one standard deviation from the mean of BMI. The analyses indicate that the main effect of commercial condition is driven by those participants with a higher BMI whose relative high food intake was significantly reduced after watching exercise commercials.

Another regression analysis of BMI on caloric intake among those participants in the control condition showed that participants with higher BMI levels had a significantly higher food intake (unstandardized beta = 10.67, p = 0.04). A similar regression analysis among participants in the exercise condition showed no significant effect of BMI on food intake. Additional bivariate correlations assessing the relationship between BMI and exercise motivations, perceived exercise behavioural control and GLTEQ showed no significant correlations (all ps > 0.20).

### Exercise variables and dietary restraint

Restrained eating was significantly positively correlated with BMI (r = 0.23, p = 0.01). A moderated regression analysis was done similar to the analysis above. Neither the main effect of restrained eating style and the two-way interaction between message condition and restrained eating style on caloric intake reached statistical significance (both ps > 0.47).

Another pathway by which commercials relating to exercise may affect food consumption is through its influence on exercise intentions and motivations. Exercise habits could also moderate the relationship between commercial exposure and food intake. Both the exercise habits of participants as measured by the GLTEQ and their estimated likelihood of going to the gym the coming week were examined for their potential moderating impact. Mean GLTEQ exercise score was 54.91 (SD = 28.43). The mean likelihood of going to the gym to have a workout was 4.42 (SD = 2.30). The correlation between these two variables was significantly different from zero (r(125) = 0.33, p < 0.001). As in the previous analyses, a moderated regression analysis was carried out, which showed no significant interaction between commercial condition and mean-centered GLTEQ (p = 0.92) on total calories consumed. As the GLTEQ specifically builds on recall of exercise habits in a typical week, it does not take into account whether participants have concrete plans to be physically active on the short term. Therefore, another moderated regression analysis was conducted with likelihood of going to the gym the coming week and commercial condition on total calories consumed. For this dependent variable, there was no main or interaction effect of likelihood of going to the gym (ps > 0.46) for total calories consumed.

## Discussion

This study was designed to understand more fully the effects of physical activity messages on food consumption. The present study demonstrated that exercise commercials can influence overall food intake. When participants were exposed to exercise commercials, their caloric intake was reduced in the meal that followed. Although the food was liked more, and despite the marginally higher ratings of hunger just before lunch after watching exercise messages, participants restricted themselves, which resulted in lowered caloric intake of 21.7%. As BMI is positively correlated with energy intake [38], we found that higher BMI levels lead to a higher food intake after watching control commercials. Importantly, we found that the overall reduction in intake was driven by participants with a higher BMI whose relative high food intake in the control condition was significantly reduced after watching exercise commercials. This finding is particularly important as it helps to explain the difference between our findings and those of Albarracin, Wang and Leeper [10] who found an increase in raisin intake after watching exercise promotion messages. Priming action goals, which is what seems to happen when individuals watch exercise commercials, may prompt a selection of active behaviours. However, which active behaviours are chosen may depend on other factors, such as the perceived ease and desirability of the action [18]. That is because people's associations with a particular topic (e.g. exercise) are based on learned responses over time building on one's history of reward and punishment with those stimuli [16]. These associative linkages between the activated concepts and related behaviours will lead to the automatic behavioural response. As indicated earlier, the perceived ease and desirability of exercise is lower for overweight individuals than for normal weight individuals [19,20,22]. The exercise commercials may have brought associations to mind which could explain the reduced intake. It is possible that the exercise commercials reminded participants of the hassle of exercise and the time it takes to burn off small amounts of calories. Accordingly, they may have felt that physical activity is a poor strategy for weight management since the energy burned up is relatively small compared to the efforts put into it. It may also be that exercise advertising leads to increased self-criticism and in this way influences food intake. In the exercise commercials in this study, ideal bodies were portrayed which for men is to gain muscle and for women to have a toned and skinny body. These ideals may have distressed overweight participants in realizing that their bodies did not fit this ideal (a contrast effect). In this respect, our results resemble the findings of Smeesters, Mussweiler and Mandel [39] who found that individuals with high BMI levels ate less and wanted to diet and exercise more after being exposed to thin models in print advertisements. Research on priming has also shown that an influence on behaviour particularly arises when an individual can identify him or herself with performance in the activated domain [17]. Nevertheless, we found no difference in response to the commercials among participants who exercised little or a lot. It could be that our sample was too homogenous in their exercise habits.

The study of Albarracin and colleagues was presented to participants as a taste task and only a very small amount of raisins were given to participants (20 each). It could be that this amount was perceived as a snack instead of a meal as in our study. Whether a person perceives a food as a meal or a snack influences what and how much one eats [40]. Furthermore, the type of messages used differed substantially between studies. Commercials showing real people actively engaged in exercise are likely to evoke different responses than cartoon-type posters as used by Albarracin and colleagues. Additionally, raisins are typically promoted and perceived as a relatively healthy snack, in contrast to a complete pasta meal with a chocolate pudding as dessert. Overall, the results of both studies raise questions that cannot yet be answered. For example, it would be useful to examine response to exercise messages based on the perceived healthiness of the food.

All participants felt more active, healthy, athletic and in good shape after watching exercise commercials compared to control commercials. Furthermore, watching exercise commercials made participants perceive the lunch as healthier and higher in liking. Provencher, Polivy and Herman [41] showed that people ate about 35% more of a snack when it was regarded as healthy than when it was seen as unhealthy. In our study, even though the food was regarded as healthier after watching the exercise commercials, this did not result in an increase of food consumption.

It is important to address the limitations of the present study. First, the study used commercials related to exercise/physical activity, which might limit the generalizability of the findings. In contrast to Albarracin and colleagues [10], we did not control for mood and arousal effects of the commercials and the exercise commercials were seen as more persuasive by the participants. Commercials may differ from physical activity campaigns sponsored by governments in several ways, i.e. the use of more youthful and attractive actors, other story lines, so the effects of exercise commercials might differ from effects of physical activity campaigns. Furthermore, we only measured participants' likelihood of going to the gym. In reality, participants may have had intentions to do other sports activities. Moreover, it is likely that the exercise commercials produced short-term reductions in food intake and that this reduction was compensated later. Hence, more research is needed to understand food consumption responses to messages related to exercise and physical activity over the long term.

Physical activity is strongly promoted because of its key role in the prevention of weight gain and obesity. Previous research suggests that physical activity and weight control messages have been minimally adopted by the public [22]. While prior research has begun to show that people's food consumption can be affected by messages about physical activity, a more thorough explanation has been lacking. Cues in the environment, such as advertising, can be enough to activate the goal of exercising. This activation may endorse related behaviours such as eating, because food intake seems tightly linked to exercise and fitness in the mind of people.

These results may have implications for the development of promotions to encourage physical activity. It is important to realize that exercise promotion messages are likely to influence more than only exercise behaviour. We found that people with higher body mass index levels are more likely to be influenced by exercise commercials. As overweight individuals are often the target of health promotion campaigns, it may be that exercise messages may lead to a food compensation mechanism and possibly even to less motivation to exercise, particularly when a promotion campaign gives people a feeling of pressure or obligation to be physically active, fit and healthy. Finally, health promotion messages aimed at increasing physical activity are unlikely to succeed unless developers fully understand the potential impact of the messages on the target audience. The results of this study provide a starting point for research into the effect of advertising promotion on food consumption, both on the short and long term.

## Competing interests

The authors declare that they have no competing interests.

## Authors' contributions

In this study, EvK conducted literature review, data analysis and wrote the manuscript. EvK and MS collected the data. MS, BW and EvK all participated in the design and coordination of the study. MS and BW helped to draft the manuscript and provided advice on data analysis. All authors read and approved the final manuscript.

## Appendix 1

### Exercise motivation

Scales used to measure exercise motivation, based on a modified version of Buckworth and colleagues' Exercise Motivation Scale [33].

'*Effort-competence' exercise motivation *(α = 0.85)

1. I think I am pretty good at physical activity

2. I put a lot of effort into physical activity

3. I am pretty skilled at the level of physical activity that I do

4. I haven't tried very hard to do well at physical activities(R)

*'Interest-enjoyment' exercise motivation *(α = 0.94)

1. I enjoy participating in physical activity very much

2. physical activity is fun to do

3. Physical activity does not hold my attention at all (reverse)

4. I think that physical activity is quite enjoyable

*'Appearance' exercise motivation *(α = 0.74)

1. I exercise to control my weight so that I look good for others

2. I don't want to look weak, so I try to work out a lot

3. I exercise so that I will not look too fat or flabby

4. people who are physically active are more attractive than those who are not

*'Competition-social' exercise motivation *(α = 0.87)

1. I play sports to win

2. My friends tell me that I am good at physical activities

3. I like a little friendly competition with my friends when I work out with them

4. My family and friends are proud of my achievements in my physical activities

### Perceived exercise behavioural control

*Perceived exercise behavioural control*, measured according to Armitage [34] (α = 0.91).

1. I am capable to participate in regular physical activity

2. I am confident that I am able to participate in regular physical activity

3. I believe I have the ability to participate in regular physical activity

### Self-esteem

Short-lived changes in self-esteem, adapted from Heatherthon and Polivy [28].

*Physical performance satisfaction *(α = 0.84)

1. I have an active life

2. I feel good about myself

3. I am confident about my physical abilities

4. I feel healthy

*Body and weight satisfaction *(α = 0.74).

1. I am pleased with my appearance

2. I am dissatisfied with my weight (R)

3. I am satisfied with the way my body looks
